# Effects of Trace Elements on the Microstructural and Machinability Characteristics of Al–Si–Cu–Mg Castings

**DOI:** 10.3390/ma15010377

**Published:** 2022-01-05

**Authors:** Yasser Zedan, Agnes M. Samuel, Herbert W. Doty, Victor Songmene, Fawzy H. Samuel

**Affiliations:** 1 Département de Génie Mécanique, École de Technologie Supérieure, Montreal, QC H3C 1K3, Canada; yasser.zedan.1@gmail.com (Y.Z.); victor.songmene@etsmtl.ca (V.S.); Fawzy-Hosny.Samuel@etsmtl.ca (F.H.S.); 2 General Motors Global Technology Center, Materials Technology, Estes Bldg, 30003 Fisher Brothers Rd, Warren, MI 48093-2350, USA; herb.doty@gm.com; 3 Département des Sciences Appliquées, Université du Québec à Chicoutimi, Saguenay, QC G7H 2B1, Canada

**Keywords:** B319.2 and 396 alloys, Sn and Bi addition, cutting forces, tool life, BUE, drilling, wear, machinability

## Abstract

This study was undertaken to emphasize the influence of Sn and Bi addition on the machinability of Sr-modified, grain-refined, and heat-treated Al–Si B319 and 396 alloys. Drilling and tapping tests were conducted to examine the cutting forces, tool life, tool wear, built-up edge evolution, and chip shape. Microstructures were examined using optical and electron microscopy. Drilling test results show that the B319.2 alloy with 0.15%Sn yields the longest drill life, i.e., twice that of the B319.2 alloy containing 0.5%Bi, and one-and-a-half times that of the B319.2 alloy containing 0.15%Sn + 0.5%Bi. The presence of 0.5%Bi in the B319.2 alloy causes a deterioration of drill life (cf., 1101 holes with 2100 holes drilled in the B319.2 alloy containing 0.15%Sn). The *α*-Fe phase in the 396 alloy produces the highest number of holes drilled compared with alloys containing sludge or *β*-Fe. The presence of sludge decreases the drill life by 50%. Built-up edge (BUE) measurements and optical photographs show little change in the BUE width for different numbers of holes except for the B319.2 alloy containing 0.5%Bi, which shows a slightly lower width (0.166 mm) compared with that containing 0.15% Sn (0.184 mm) or 0.15%Sn + 0.5%Bi (0.170 mm).

## 1. Introduction

**Figure 1:**
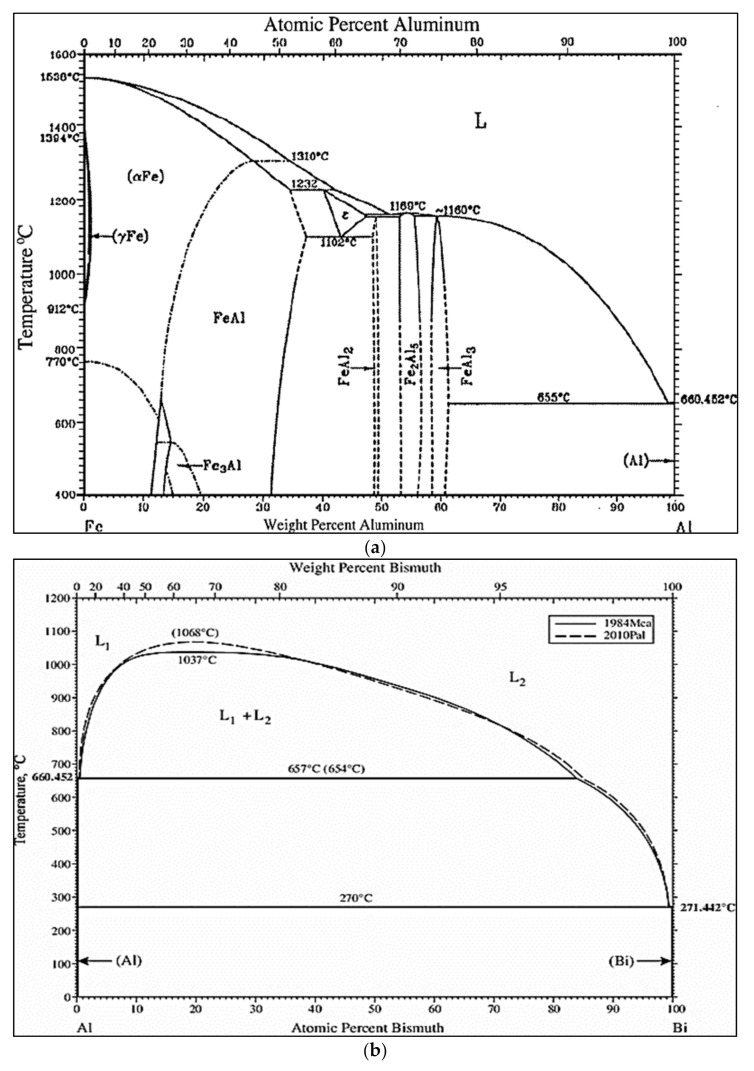

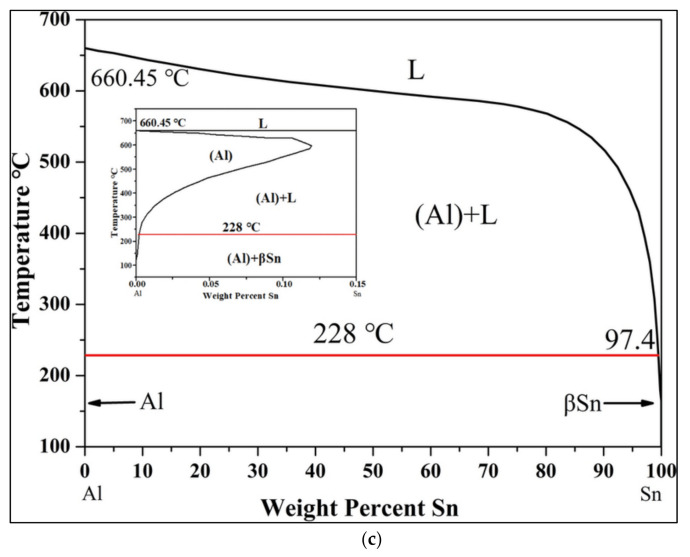
Binary phase diagrams for (**a**) Al–Fe [1], (**b**) Al–Bi [2], and (**c**) Al–Sn systems.

Considering machinability as a system property allows us to define machinability as an interaction phenomenon between the workpiece (with different alloying elements such as Fe, Cu, Mg, and Mn), cutting tool (tool material and geometry), and cutting medium (wet or dry cutting) for different removal sequences such as turning, drilling, tapping, milling, and sawing under different cutting conditions, e.g., cutting speed, feed rate, and depth of cut [1,2,3,4,5]. For example, the presence of Fe in an Al alloy can lead to the formation of different intermetallic phases, as shown in Figure 1a. The presence of these hard phases can affect the machinability of the workpiece.

One of the approaches for improving machinability is the use of free-cutting alloys. These alloys were developed from standard heat-treatable alloys, to which were added elements for forming additional phases in the aluminum (Al) matrix [6]. Free-cutting constituents are formed in the presence of alloying elements with the following properties: (a) insolubility in both liquid and solid Al; (b) a low melting point with regard to Al; (c) not forming intermetallic compounds with Al or other alloying elements; and (d) having lower hardness values compared with the Al matrix. These conditions may be fulfilled by using lead (Pb), bismuth (Bi), tin (Sn), cadmium (Cd), indium (In), antimony (Sb), or a number of other elements that are, however, unusable from the practical point of view. These lubricity-imparting additives are not entirely soluble in the solidified Al-rich matrix phase of the castings, although they may combine with such alloying constituents as magnesium (Mg). They appear dispersed as small globular bodies in the cast metallurgical microstructure [6,7,8,9,10]. According to Young [11], Bi and/or Sn are the additives preferred by most researchers [12]. It should be remembered that if the amount of the additive is not high enough, the low melting point constituents would need to be well dispersed to have any significant impact on machinability [13].

The lower strength values of the alloys containing added Sn result from the distribution of soft fine particles of β-Sn, mainly on the grain boundaries, as well as from the formation of other intermetallic compounds such as Mg_2_Sn [14]. Tin and In, to a certain extent, are used most frequently as substituents. Smolej et al. [15] demonstrated that Sn together with phases based on Al and Cu formed nets enveloping the crystal grains of the matrix, although Sn was not found in the crystal grains themselves with regard to as-cast billets of AlCuMgSn alloys containing only Sn for the improvement of machinability. The size and distribution of free-cutting constituents in the matrix are important factors in the machinability of alloys.

Akyüz [16] reported that cutting efficiency was improved in Al-13%Si alloys containing 0.2~0.4%Sn; thus, the cutting resistance was decreased, the cutting temperature was lowered, and the progress of tool wear was retarded. The improvement in machined surfaces was not clearly defined, although the treatment of chips became easier because their shape had changed to a sheared form. On the other hand, in Al-23% Si alloys containing the same level of Sn, i.e., about 0.2–0.4%, the improvement in machinability was not significant. Neither the decrease in cutting force nor the drop in cutting temperature was at all obvious. The effects of the addition of Sn on tool wear and machined surfaces were also not distinct. The chip shape of the original alloy was likely to be in sheared form, and the alloy containing Sn produced finer chips of a similar shape.

Additions of Bi produce continuous self-lubricity and provide low friction and resistance to high seizure loads. According to the Al–Bi phase diagram [1], Al and Bi dissolve completely in each other at temperatures above 1037 °C, thus forming a homogeneous melt. Below this temperature, however, there exists a miscibility gap in the liquid state. An Al-rich melt coexists with a Bi-rich melt, which has the higher density. At 657 °C, the Al-rich melt decomposes into a solid Al and Bi-rich melt, i.e., it produces a monotectic reaction at 3.4%Bi, whereas at 237 °C, the Bi-rich melt itself solidifies (Figure 1b). Figure 1c presents the Al–Sn binary phase diagram [17].

It should also be noted that the solubility of Bi is less than 0.24 wt% at the monotectic temperature, whereas the limit of its solubility in Al at the eutectic temperature is assumed to be almost nil [1]. Bismuth appears to have significant potential as a nontoxic alternative to Pb for enhancing the machinability of Al alloys because of the similarity of features between Pb and Bi, e.g., a low solubility in Al alloys and a low melting point. It was assumed that an increase in the number of Bi particles, which in fact also implies a decrease in the mean spacing of these particles, could be considered more effective in producing fine chips than would be an increase in the Bi content. The reduction in mean spacing of Bi particles is achieved by a refinement in the α-Al grains of the matrix attributed to the high wettability of Bi at the grain boundaries [18].

Bismuth, a highly reactive element, reacts not only with Sr, Na, and Ca, which themselves contribute to the modification of the Si-phase, but also with Ni and Mg to increase the strength of the alloy so that the functions of these elements decrease as do the mechanical properties of the alloy. Loper [19] demonstrated that Bi in Al–Si alloys interacts with major modifiers, such as Na and Sr. He postulated that the formation of a ternary Bi–Mg–Sr compound (Bi_2_Mg_2_Sr) and/or binary Bi–Sr compounds (Bi_3_Sr, BiSr, Bi_2_Sr_3_, and BiSr_2_) would occur as a result. The Bi-phase, however, does not form an intermetallic compound with Al because it is independently distributed [8,19,20,21].

Limited research has been carried out on the effects of free-cutting elements on the machinability of Al–Si casting alloys. The current study addresses this point by investigating the influence of individual and combined additions of Sn and Bi on the machinability of Sr-modified, grain-refined, and heat-treated Al-(7–11)%Si alloys, where the machinability criteria relate to tool life, tool wear, evolution of the BUE, cutting forces, and moment analysis.

## 2. Materials and Experimental Procedures

The B319.2 and 396 base alloys (Table 1) used here were supplied in the form of 12.5-kg ingots that were cut into smaller pieces, dried, and melted in charges of 100 kg each for the preparation of the various alloy compositions. Melting was carried out in a SiC crucible of 120 kg capacity using an electrical resistance furnace in which the melting temperature was maintained at 750 ± 5 °C. At this temperature, measured amounts of Sn (as the pure metal), Bi (as the pure metal), strontium (Sr) as the Al-10%Sr master alloy, and titanium (Ti) as the Al-5%Ti-1%B master alloy were added to obtain the desired levels. The melts were degassed using pure dry argon injected into the melt by means of a rotary graphite impeller rotating at 150 rpm for a duration of 15–20 min. It should be noted that, for all the castings involved, the humidity level was between 18% and 20%. Due to the high affinity of Sr to react with oxygen, Sr was added only 5 min before pouring. In addition, small amounts of Sr (~20% of the original concentration) were added after every 5 blocks that were cast. Surface oxides and inclusions were skimmed off thoroughly prior to pouring. For each alloy, 15–20 blocks were cast. The melt was poured at ~740 °C into a preheated (450 °C) waffle-plate graphite-coated metallic mold to prepare the castings for machinability studies. Samplings for chemical analysis were also taken concurrently for each melt condition. The chemical compositions and the respective codes for the various alloys prepared from the base B319.2 and 396 alloys as determined by optical emission spectroscopy are listed in Table 2. About 60 blocks were cast for the present study.

The heat treatments were selected in such a way as to establish the hardness level as a common factor for all the alloys studied, within the range of 110 ± 10 BHN. This range is the one most frequently used in the majority of commercial applications for aluminum alloys. Solution heat treatments were carried out at 490 °C/8 h for the B319.2 and 396 alloys, which were then quenched in warm water at 65 °C, followed by artificial aging at 200 °C for 5 h (i.e., T6 tempered). Both solution and aging heat treatments were carried out in a forced-air electric furnace with a programmable temperature controller accurate to within ±2 °C.

Samples measuring 25 mm × 25 mm for metallographic examination were sectioned from the machinability test sample and mounted in bakelite using a Struers Labopress-3 Mounting Press (Spectrographic Limited, Leeds, UK). These samples were then ground and polished to the desired fine finish on 9, 6, 3, and 1 µm diamond lap wheels. After that, the microstructure was examined with an Olympus PMG3 optical microscope (Leco microscopes, Salem, MA, USA). The eutectic Si particle characteristics, including particle area, length, roundness (%), aspect ratio, and density (particles/mm^2^), were measured and quantified using a Clemex image analyzer system in conjunction with the optical microscope. For each sample, 50 fields were examined at a magnification of 500×, in such a way as to cover the entire sample surface in a regular and systematic manner. A Hitachi SU-8000 field emission scanning electron microscope (FESEM) (Hitachi High-Technologies Corporation, Tokyo, Japan), equipped with a standard secondary electron detector (SE), a backscatter electron detector (BSD), and an energy dispersive X-ray spectrometer (EDS), was used for examining the fracture surface.

Hardness measurements were carried out on the as-cast and heat-treated samples using a Brinell hardness tester, applying a steel ball of 10 mm diameter with a load of 500 kg exerted for 30 s. Four sample blocks were used for each alloy condition. The reported hardness values represent the average of at least 160 indentation readings, namely, 40 indentations total per block on both sides of the opposite surfaces of the five ribs of the waffle-plate casting block. The hardness results for selected alloys are listed in Table 3.

**Figure 2:**
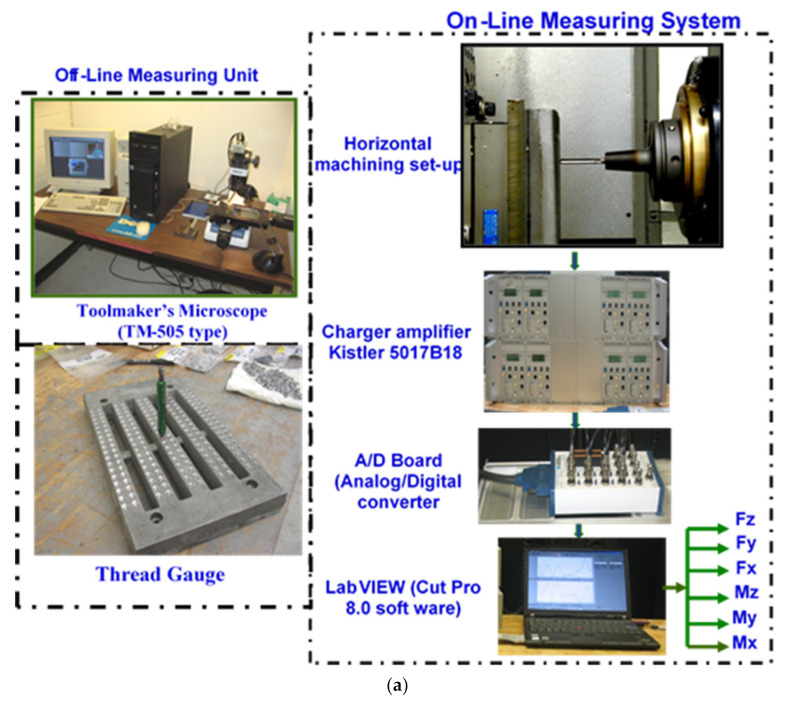

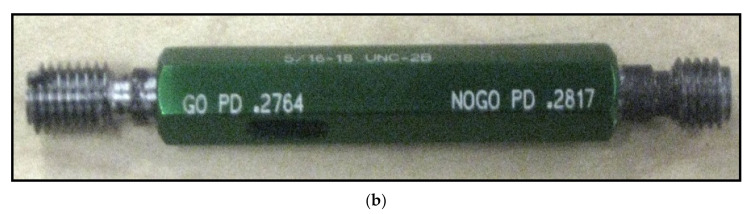
(**a**) Experimental setup for carrying out the drilling and tapping tests, (**b**) Thread gauge used for conducting Go/No-Go gauge tests.

The specimens were cut from the waffle-plate casting having the overall dimensions of 300 mm length, 175 mm width, and 30 mm thickness, with ribs approximately 25 mm wide, separated by gaps of 16 mm, as shown in the photograph at the bottom left in Figure 2. Machinability tests were carried out using a Makino A88E (Makino Milling Machine Co., Ltd., Tokyo, Japan) high-speed horizontal machining center operating at 30 kW power and a maximum rotation speed of 18,000 rpm. The experimental setup consisted of a dynamometer with four sensors, charge amplifiers, and an A/D converter; it was used for the online measurement of drilling and tapping forces, as shown in Figure 2a, while a toolmaker’s microscope was used for observing and recording the built-up edge (BUE) measurements and tool wear characteristics.

A Go/No-Go gauge test was carried out after the drilling and the tapping of each test block to check the dimensional accuracy for both drilled and tapped holes. Figure 2b displays the thread gauge that was used for conducting the Go/No-Go test. Reference diameters of 6.5024 to 6.5278 mm and 7.02056 to 7.15518 mm from the Go/No-Go gauge test were applied to the drilling and tapping processes, respectively [14].

As a rule, cemented carbides of K-type quality are used for the short-chipping of Al-Si alloys. The drills employed for the purposes of this study were made of an uncoated ISO K20 carbide or RT 150 GG in accordance with Guhring specifications. A straight flute and coolant-fed carbide “G” drill with an average diameter of 6.5 mm and a 90 mm length was used to drill two rows of holes on each rib of the waffle-plate with 4 mm between rows. Conventional TiN-coated HSS cutting taps with a specification of M8*1.25-6H and three spiral flutes were used in subsequent procedures in the tapping process. Tapping was carried out immediately after drilling for the drilled holes over the full plate. The cutting conditions for drilling and tapping are listed in Table 4 [14]. Since increasing temperatures and the potential accumulation of hot chips at the bottom of the hole tend to present serious problems, a pressurized coolant was pumped through the drill to ensure adequate cooling and chip evacuation. A synthetic metalworking fluid concentrate, CIMTECH^®^ 310 (composed of 5% cutting fluid + 95% liquid), was applied to avoid the effects of any heat that might be generated during machining.

In this study, each alloy was tested with a new drill. If the drill broke during the drilling process, one of two options was selected: (i) drilling was halted, then the test was transferred to another series; or (ii) in the case when the drill broke due to the presence of a defect or large inclusion, the test was resumed on the same casting block using a new drill. In the case of the tapping process, failure is said to occur either when the drill fails or the tap gauge no longer goes into the hole. The width of the built-up edge (BUE) on the tool was also measured in the middle and at the end of each test period of the sample casting; this was carried out using a toolmaker’s microscope (TM-505 type) at a magnification of 50×.

In order to follow the development of cutting-tool wear, the forces involved in the cutting process were also monitored. A 6-component piezo-electric quartz crystal dynamometer, Model-9255B, from Kistler Instruments (Kistler Instrument Corp., Novi, MI, USA), was used for the online measurement of the forces and moments in drilling and tapping (Fx, Fy, Fz, Mx, My, and Mz). A charge amplifier, Type-5017B18, with eight independent measuring channels was used for the measurement of the combined force-and-moment data input. Drilling moment (Mz) and feed force (Fz) are of particular interest in analyzing the drilling process. The deflective forces Fx and Fy perpendicular to the rotary axis provide additional information on the machining process.

## 3. Results and Discussion

### 3.1. Microstructure

**Figure 3:**
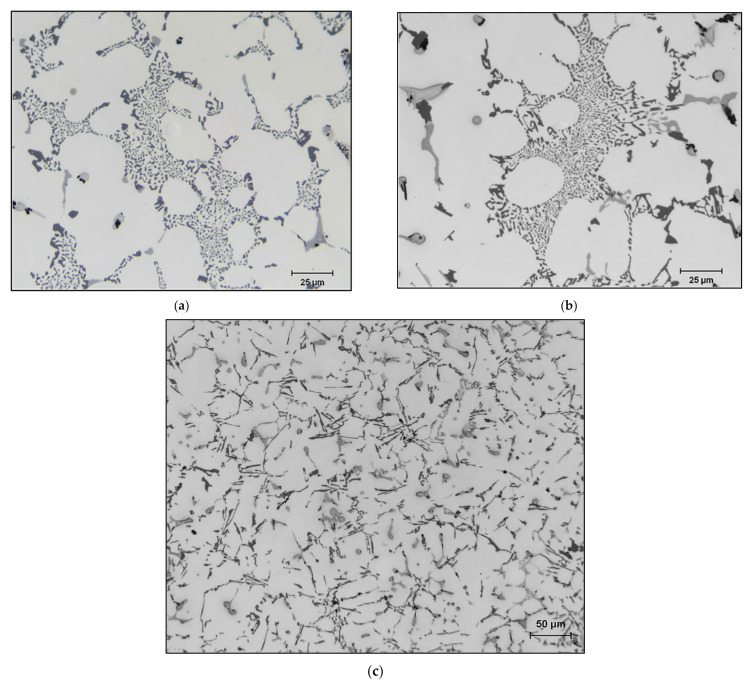
Optical microstructures of (**a**) the 319.2 base alloy, (**b**) the M12 alloy, and (**c**) the M13 alloy in the as-cast condition.

Figure 3a depicts the microstructure of the base alloy B319.2 in the as-cast condition, revealing a well-modified structure (Sr content ~ 150 ppm). Addition of 0.15%Sn to the base alloy, i.e., M12 alloy in the as-cast condition (Figure 3b), reveals a microstructure similar to that shown in Figure 3a. However, this was not the case when 0.52%Bi was added to the base alloy, resulting in a completely un-modified Si structure (M13 alloy) as shown in Figure 3c.

**Figure 4:**
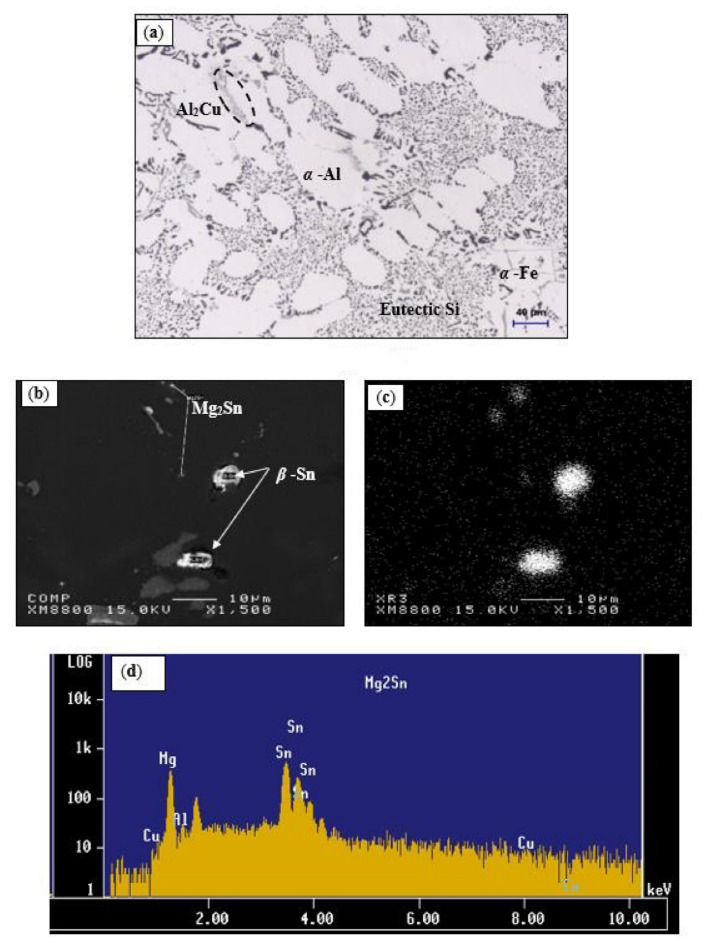
(**a**) Optical microstructure of the base 396-M1 (Al-11%Si) alloy in the as-cast condition; (**b**) high magnification backscattered image of the M2 alloy showing precipitation of β-Sn; (**c**) X-ray image of Sn distribution in (**b**); (**d**) EDS spectrum revealing intensive reflections due to precipitation of Mg_2_Sn phase particles in (**b**).

The microstructure of the Sr-modified, grain-refined 396-M1 (Al-11%Si) alloy in the as-cast condition is shown in Figure 4a. Eutectic silicon particles that precipitated in the form of fibrous crystals typical of a modified structure represent the main microstructural feature observed. It will also be noted that the iron precipitates in the form of the coarse α-Fe phase, which is always seen to occur within the α-Al dendrites, indicating that it corresponds to a pre-dendritic reaction, and thus tends to nucleate in the liquid alloy prior to solidification. The copper phase, on the other hand, may be seen mainly as small pockets of the block-like Al_2_Cu phase nucleating either within the aluminum matrix or at the interface of such pre-existing constituents as Si or intermetallic particles. The platelet-like β-Fe phase was not in evidence because of the higher Mn/Fe ratio in the 396-M1 alloy (~1.17); this ratio promotes the formation of the α-Fe phase at the expense of the β-Fe phase.

Figure 4b shows a backscattered image, obtained at high magnification, of the heat-treated 396-M2 alloy containing 0.15%Sn, in which the precipitation of Sn in the form of white phase particles may be observed. These white particles are β-Sn. The distribution of the β-Sn particles is not uniform; rather, they appear as small clusters. This figure shows that the tin also precipitates as tiny Mg_2_Sn particles on the eutectic Si particles; high Sn concentrations may be observed in Figure 4c for the β-Sn particles shown in Figure 4b. The EDS spectrum in Figure 4d shows reflections due to the Mg_2_Sn particles observed in Figure 4b, while Table 5 presents the chemical composition of these particles.

**Figure 5:**
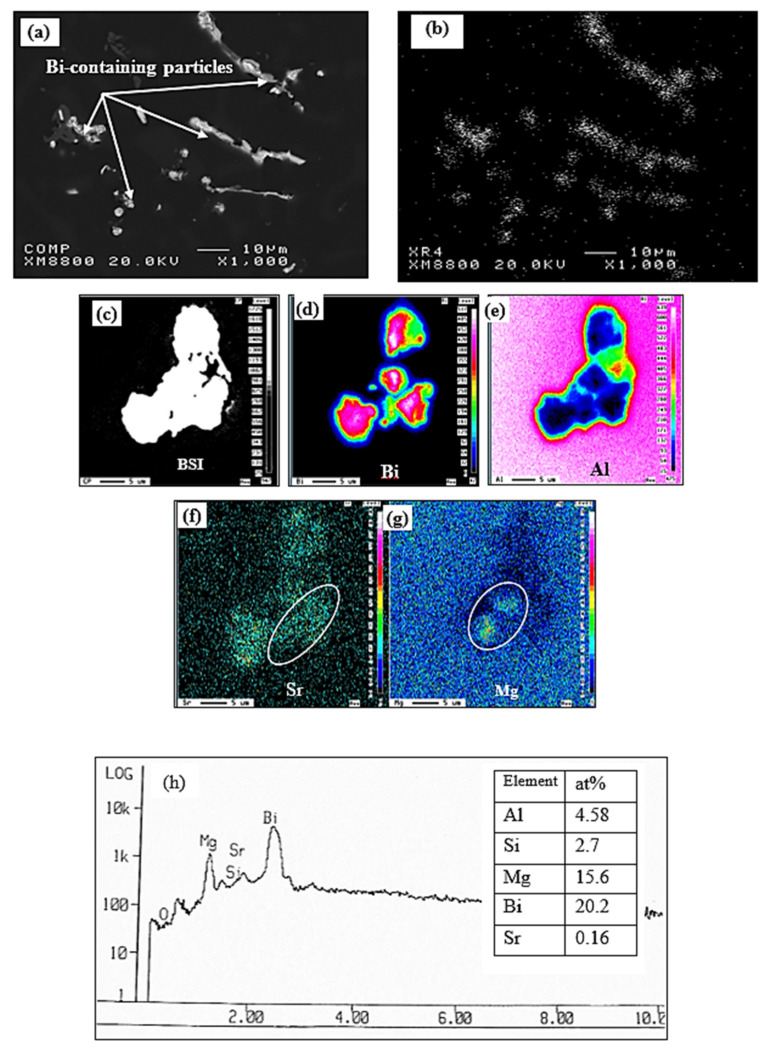
(**a**) Backscattered electron image of the Bi distribution with Al-matrix in M7 alloy, (**b**) X-ray image of the Bi distribution in (**a**), (**c**) backscattered image of Bi-containing particles, (**d**–**g**) X-ray images of the Bi, Al, Sr, and Mg distribution in the Bi-containing particles, and (**h**) EDS spectrum showing an example of reflections due to Bi, Sr, and Mg [23].

The size of the bismuth constituents in the Al–Si alloy matrix vary to a certain extent; thus, a sufficiently fine distribution is preferred so that free machining prevails throughout the work piece. A preferred range for the size of the Bi particles is up to about 10 microns, and even more suitably, up to about 5 microns [19]. Figure 5a,b provide the backscattered image and X-ray image of Bi, respectively, for the 396-M7 alloy containing 0.5%Bi. Figure 5b shows that the Bi-phase is not uniformly distributed in Al–Si alloy structures and that this phase tends to segregate and form a coarse phase because of the high wettability of Bi at the grain boundaries.

Figure 5c shows a backscattered image of a cluster of Bi-containing particles, where the corresponding X-ray images of Bi, Al, Sr, and Mg displayed in Figure 5d through Figure 5g reveal that these particles contain a negligible amount of Al, suggesting that they are likely Bi_2_Mg_2_Sr, as reported by Blachnik [22]. Figure 5h is an example of elements in a Bi–Mg–Sr compound [23].

### 3.2. Silicon Particle Characteristics

**Figure 6:**
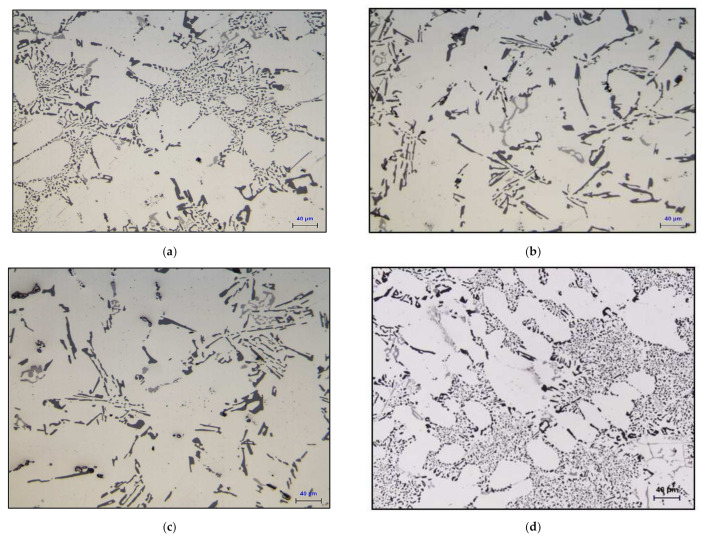

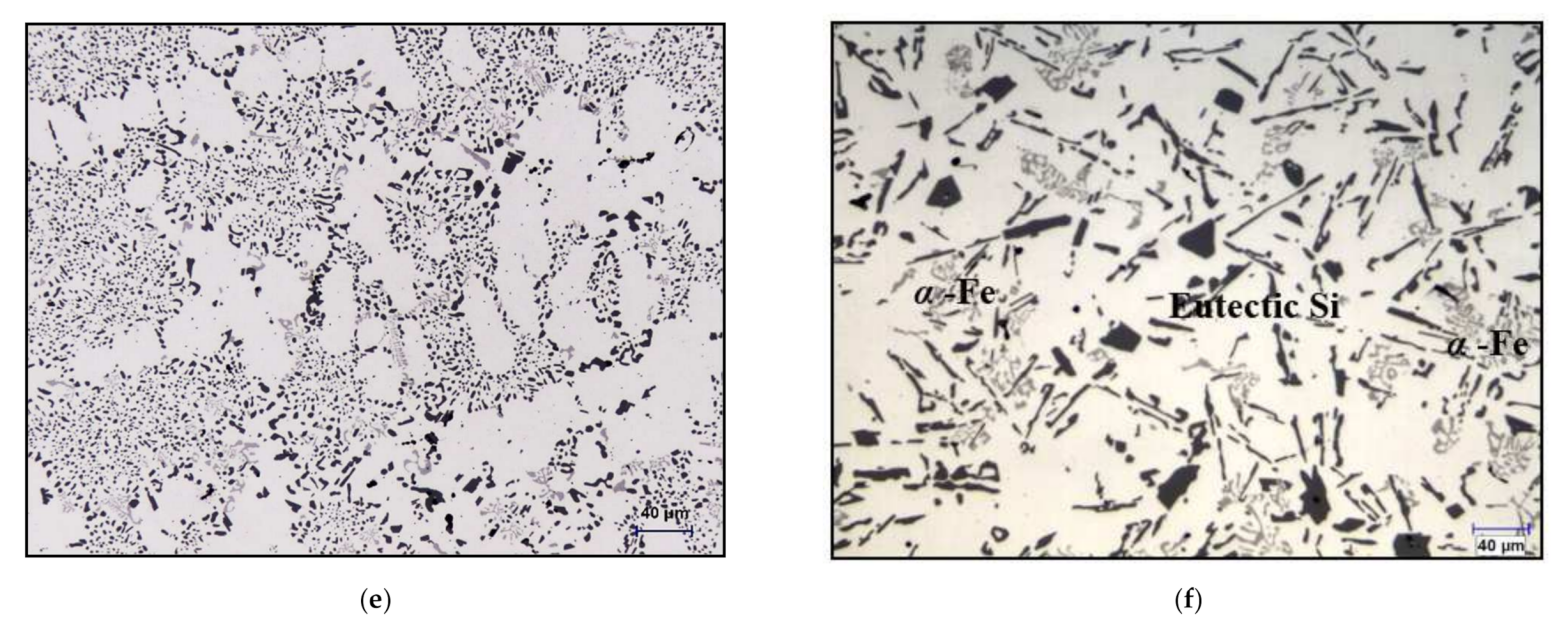
Optical micrographs showing the effect of Bi and Sn addition on the microstructure of heat-treated B319.2 and 396 (Al-11%Si) cast alloys. (**a**) M12 (B319.2 + 0.15%Sn), (**b**) M13 (B319.2 + 0.5%Bi), (**c**) M14 (B319.2 + 0.15%Sn + 0.5%Bi), (**d**) M1 (396), (**e**) M2 (396 + 0.15%Sn), (**f**) M7 (396 + 0.5%Bi).

Figure 6 shows the effect of the Bi and Sn additions on the microstructure of the Sr-modified, grain-refined, and heat-treated 396 (Al-11%Si) alloys. Table 6 summarizes the eutectic Si particle characteristics obtained for the alloys investigated. It may be observed that the addition of 0.15%Sn to the Al-(7–11)%Si alloys leads to a slight coarsening of the eutectic Si particles, as seen in Figure 6a,e; for example, the average roundness and density decrease by 7% and 18%, respectively. The addition of 0.5%Bi to the Al-(7–11)%Si alloys counteracts the modifying effect of Sr, as shown in Figure 6b,f, leading to a noticeable coarsening of the Si crystals. The eutectic Si particle analysis of the average particle area showed a jump in this parameter from 4.3 µm^2^ in the 396-M1 alloy to 49 µm^2^ in the 396-M7 alloy. Additionally, the particle length increased from 2.96 µm in the M1 alloy to 15.3 µm after the addition of Bi. The degradation of Sr modification in the presence of Bi may be interpreted in terms of a Bi–Mg–Si interaction and/or Bi–Sr interaction. It is possible that a ternary compound (Bi_2_Mg_2_Sr) and/or the binary compounds (Bi_3_Sr, BiSr, Bi_2_Sr_3,_ and BiSr_2_) may form, resulting in a reduction in the amount of Sr available for modification. The effect of Bi addition is clearly evidenced in the noticeable demodification of the Si particles in the Sr-modified 396-M1 alloy at about 0.5%Bi addition, as seen in Figure 6f.

### 3.3. Cutting Forces

Table 7 provides the relevant data relating to T6 heat-treated mechanical properties; these include the hardness (BHN), yield strength (YS), ultimate tensile strength (UTS), and percent elongation (%El) of the Sr-modified 396 alloys after the addition of Sn and Bi [14]. The corresponding hardness data indicate that the M7 alloy containing 0.5%Bi has the highest hardness values compared with the other alloys. On the other hand, the presence of 0.5%Bi in the M7 alloy results in a decrease in the YS, UTS, and %El by 10%, 9%, and 34%, respectively. The significant reduction observed in the ductility value of the M7 alloy may be attributed to the effects of the addition of Bi in transforming the structure of the M1 base alloy from a well-modified to a non-modified structure, as shown earlier in the discussion on microstructure. It is important to mention here that the reduction caused in the tensile properties of the M7 alloy, compared with the M1 alloy, may be explained in terms of the Bi–Mg interaction, which consumes the Mg available for the precipitation of Mg-hardening phases. It was reported that Bi is a surface-active element, and its accumulation near intermetallic and Si particles may reduce the energy of the system. Most of the Bi particles were connected to intermetallic phases and Si phases, which has a deteriorating effect on the mechanical properties of the alloys. It will be observed that the addition of 0.15%Sn causes a marginal reduction in either the hardness or the tensile properties of the 396-M1 alloy.

(a) Alloy 396 (Al-11%Si)

The microstructure of the 396 (Al-11%Si) alloy is eutectic and composed of soft (α-Al) and brittle phases. The brittle phase consists of acicular silicon distributed in the eutectic microstructure. Chip formation is related to the capacity of the material for plastic deformation, the fractions of the soft and brittle phases in the microstructure being very important. In the plastic deformation of this alloy, a uniform and fine distribution of the soft phase and silicon needles limiting the plastic deformation of the soft phase is equally important. As the Al-11%Si alloy consists of a smaller fraction of the soft phase and, consequently, of a larger fraction of the brittle phase with reference to the Al-7%Si hypoeutectic 319.2 alloy, the zone of the microplastic deformation in the cutting zone obtained under the same cutting conditions is thus considerably reduced. The total mean cutting force and moment together with the corresponding standard deviation bars based on the mean value of 100 holes, i.e., 40 holes per rib, were used as a way to evaluate the machinability criteria of the alloys studied.

**Figure 7:**
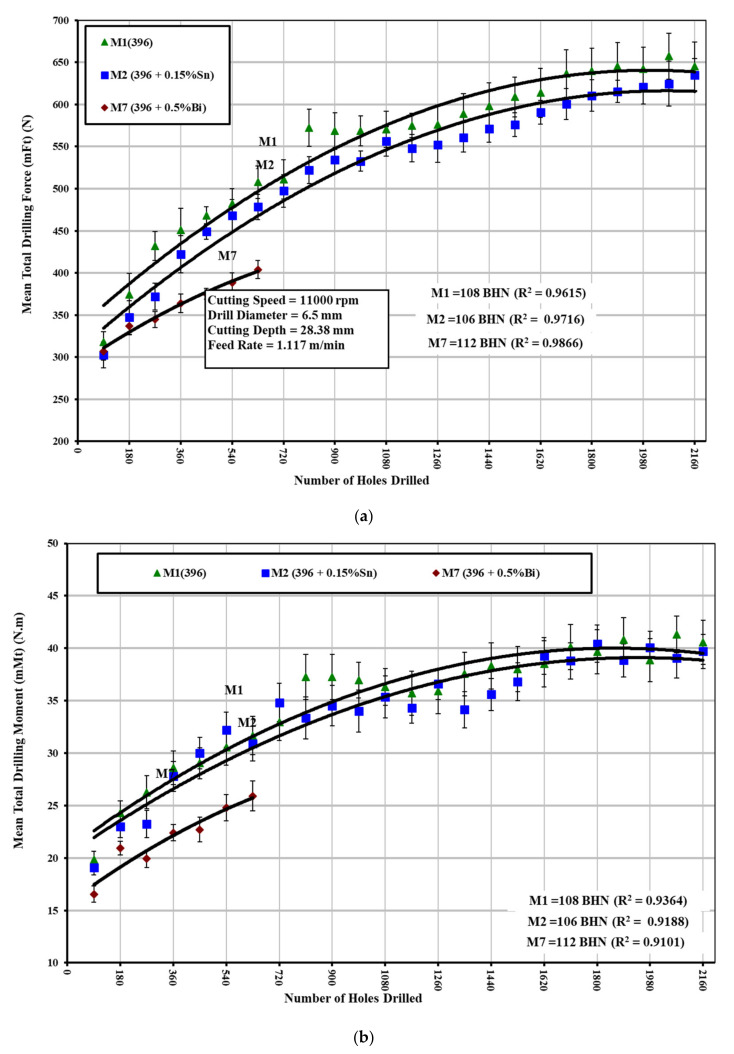
Effect of Sn and/or Bi additions on the machinability of Sr-modified 396 (M1, M2, and M7) alloys in terms of: (**a**) average total drilling force; and (**b**) average total drilling moment of 100 holes.

**Figure 8:**
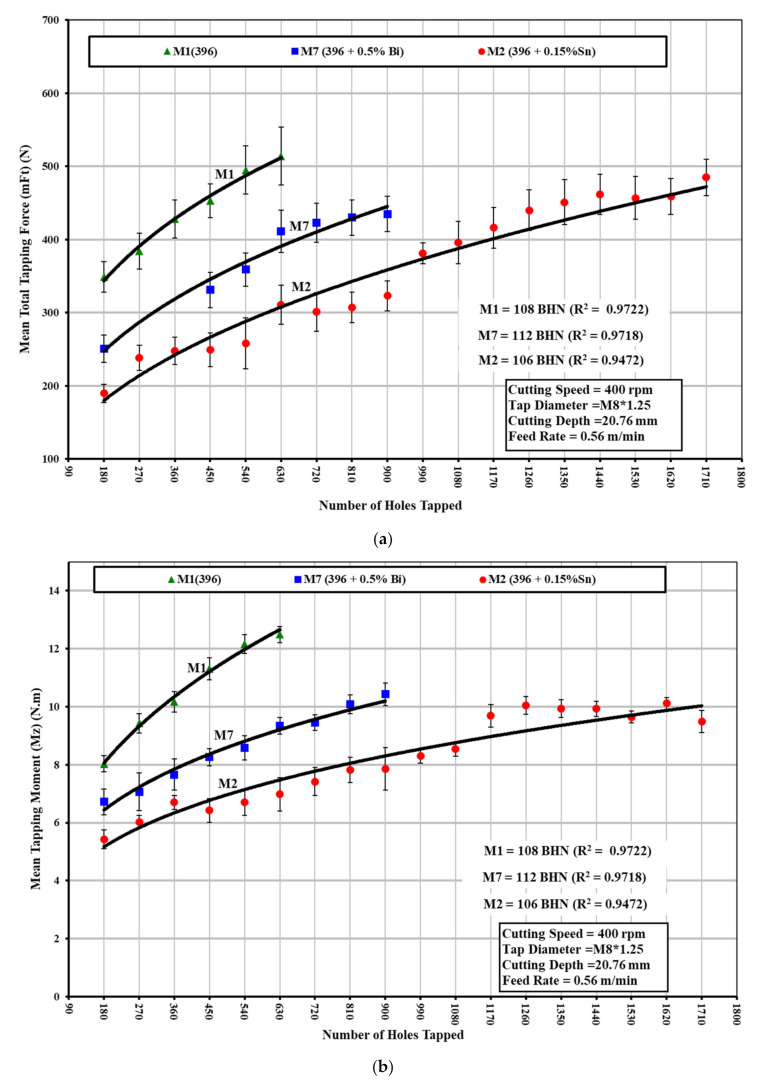
Effect of Sn and/or Bi additions on the machinability of Sr-modified 396 (M1, M2, and M7) alloys in terms of: (**a**) average total tapping force; and (**b**) average total tapping moment of 90 holes.

A number of Sr-modified, grain-refined, and T6 heat-treated 396 alloys were selected in order to study the effects of Sn and Bi addition on the drilling and tapping forces and moment of the 396-M1 base alloy, the 396-M2 alloy containing 0.15%Sn, and the 396-M7 alloy containing 0.5%Bi and are shown in Figure 7 and Figure 8. The experimental results from the drilling test reveal that the addition of 0.5%Bi to the 396-M7 alloy has a significant influence in decreasing the drilling forces and moment compared with the 396-M1 base alloy. The effect of 0.15%Sn addition to the 396-M2 alloy on the drilling forces and moment is not obvious, as may be seen clearly in Figure 7a,b. A noticeable reduction in cutting forces by means of the addition of Bi may be obtained either by softening of the Bi particles as a result of an increase in the local temperature or by void formation due to a deformation mismatch between Bi and the Al matrix during machining. It is also possible that a combination of both these processes is at play during machining, which gives rise to an improvement in machinability.

During the tapping process, conventional TiN-coated HSS taps with a relatively low cutting speed and feed rate were used (compared with the cemented carbide drills and high cutting speed and feed rate used in the drilling process). The experimental results from tapping tests show that the presence of 0.15%Sn in the 396-M2 alloy has a beneficial effect on decreasing the cutting forces and moment compared with the 396-M1 base alloy, as shown in Figure 8a,b. The size and distribution of the free-cutting element in the matrix are important factors in the machinability of this alloy. The Sn phases were observed to be smaller and more densely distributed compared with the Bi-containing particles, as shown in Figure 4.

(b) Alloy B319.2 (Al-7%Si)

**Figure 9:**
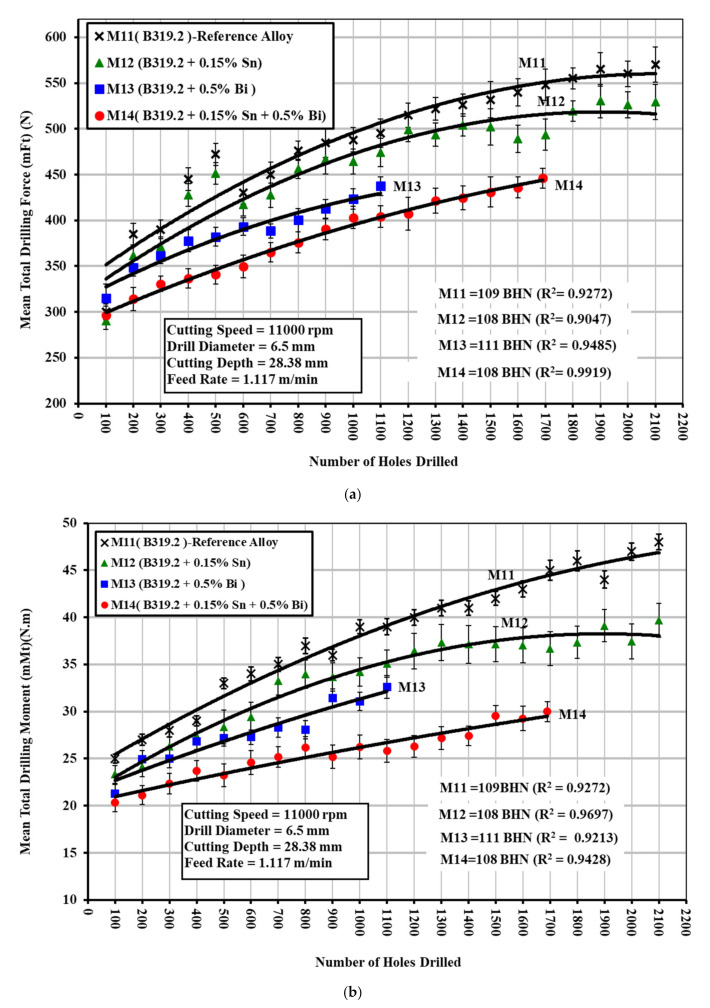
Effect of Sn and/or Bi addition on the machinability of B319.2 (M11), M12, M13, and M14 alloys in terms of (**a**) mean total drilling force; and (**b**) mean total drilling moment for drilling 100 holes (40 holes per rib).

The alloys in question were represented by B319.2 + 0.15%Sn, coded M12; B319.2 + 0.5%Bi, coded M13; and B319.2 + 0.15%Sn + 0.5%Bi, coded M14, yielding hardness values of 108, 111, and 108 HBN, respectively. The results from the drilling tests reveal that the presence of 0.5%Bi in the B319.2-M13 alloy improves the alloy’s machinability by lowering the drilling force and moment by 19% and 22%, respectively, compared with the B319.2-M12 alloy containing 0.15%Sn, as shown in Figure 9a,b. The causes of, or the mechanisms involved in, the reduction in cutting forces for the alloy containing 0.5%Bi may be any of the following: (i) an effect related to void formation due to non-uniform deformation of Bi and the Al matrix during the machining process; (ii) an effect related to Bi being a low-melting-point constituent as compared with the aluminum base alloy; (iii) a combination of both. With regard to the first effect, since Bi is elemental and does not occur in the form of a eutectic or low-melting-point compound, it can persist as a discontinuity in the aluminum alloy matrix.

Consequently, when the matrix material is being subjected to machining forces, the Bi will tend to react to shearing stresses in a different manner with respect to its surrounding aluminum alloy matrix, thereby creating voids between them. The continued application of machining forces creates new voids and propagates existing ones until these voids interconnect and machining chips are formed. With regard to the second effect, Bi may act as a low-melting-point constituent during machining, when the bismuth may soften or melt as a precursor to breakage, chip formation, and material removal, since the melting point of bismuth is 271 °C, while that of the aluminum base is 660 °C. Additionally, during machining, a combination of all the effects may occur and lead to a reduction in the cutting forces and moment for this alloy.

The simultaneous addition of smaller amounts of two or more elements insoluble in aluminum has a higher effect on machinability than individual additions of each element in terms of cutting forces and moment. Addition of 0.15%Sn plus 0.5%Bi to the B319.2-M14 alloy lowers the drilling forces and moment by 22% and 27%, respectively, compared with B319.2-M12 containing 0.15%Sn, as may be seen in Figure 9a,b. This improvement may be attributed to the fact that the chip breaking of aluminum alloys is a consequence of heating of the Sn and Bi particles at temperatures generated within the material during the cutting process. Heating increases the volume of both these elements at the expense of the matrix. It is presumed that the Sn and Bi phases will become incipiently fused at cutting temperatures. The stresses generated as a result of the increase in volume and the incipient fusion of these phases, which may lead to notch effects, tend to decrease the ductility of the material in the cutting zone, causing a reduction in cutting forces. These results are consistent with work carried out by Smolej [21], which confirmed that tin phases together with other phases of alloying elements and impurities reduced the cohesion of crystal grains, thereby leading either to the accelerated rupture of the material during the course of cutting or to easier breakage of chips.

The tapping process is related to the multi-flute/multi-land engagement between the cutter (tap) and the hole (work piece). The chamfer section of the tap generates a major part of the resulting cutting force, mainly because of the larger chip cross-section, which is related to the teeth of the chamfer, while machining with a calibration section does not contribute significantly to the resulting force because friction effects are dominant throughout this phase. During the tapping test, tap breakage was observed frequently and since microscope inspections of the taps did not indicate any severe wear on the cutting edges, it was concluded that the tool breakage was not a result of tap wear. An examination of broken taps indicated that the breakage mode was torsional, from which it was reasoned that chip-clogging loads exert excessive torque on the tap.

**Figure 10:**
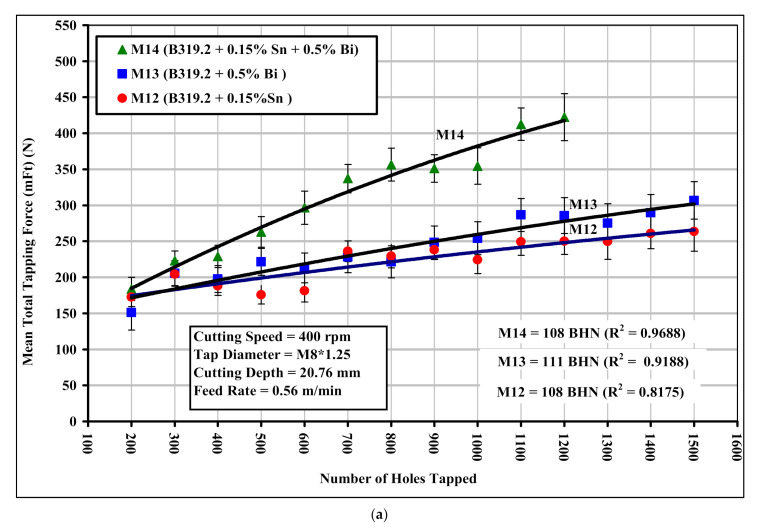

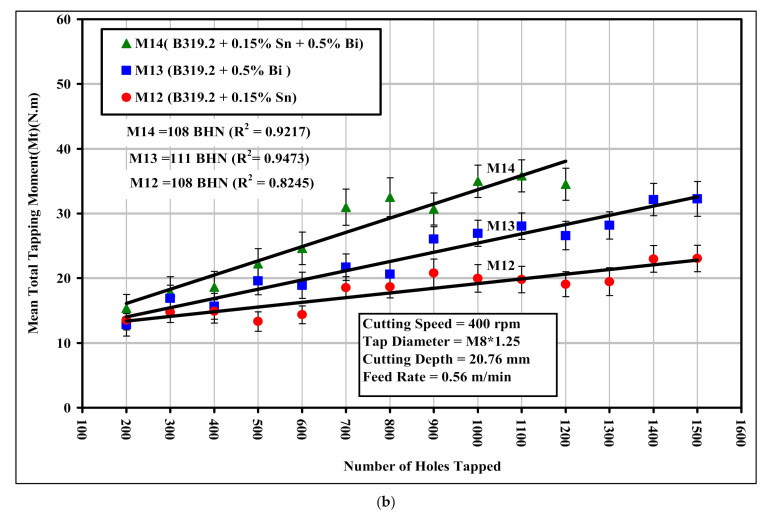
Effect of Sn and/or Bi addition on the machinability of B319.2-based M12, M13, and M14 alloys in terms of (**a**) mean total tapping force; and (**b**) mean total tapping moment required for tapping 100 holes (40 holes per rib).

As a result of such difficulties, the normal cutting speeds used in tapping operations on B319.2 and 396 alloys are relatively low, i.e., around 10 m/min (400 rpm), compared with the speeds involved in the drilling process, i.e., around 234 m/min (11,000 rpm). As may be seen clearly in Figure 10a,b, the addition of 0.15%Sn + 0.5%Bi to the B319.2-M14 alloy increases the tapping force and moment by 61% and 72%, respectively, compared with the B319.2 alloy containing 0.15%Sn. The total tapping load consists of a base load and a chip-packing load, which is the result of chip clogging in the flutes. The chip-packing load may be as much as five times the base load. The increase in cutting forces and moment may thus be explained by the fact that the breaking of chips is best at the eutectic composition of those elements that are insoluble in aluminum; there was, however, no evidence for the formation of any eutectic compound between Bi and Sn in the B319.2-M14 alloy.

### 3.4. Tool Life

In a collective sense, the most important parameters that relate to the life of the tools used in drilling and tapping modes (which were investigated in this study) are:

- Specific power consumed, cutting force, moment, and tool life; and
- Heat build-up, hole accuracy, and chip control (chip breakability)

**Figure 11:**
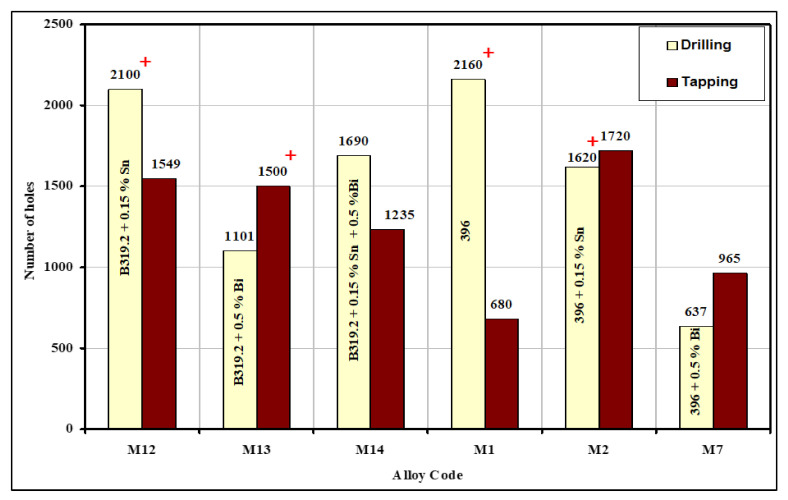
Comparison of tool life in terms of number of holes drilled and tapped in Sr-modified B319.2 and 396 alloys, containing additions of different alloying elements. The symbol (+) indicates that the drill was unbroken.

In a drilling operation, the performance criterion is judged by drill life, expressed as the number of holes to failure or the equivalent drilling time. Failure is usually defined either by the sudden occurrence of a violent noise or by the catastrophic fracture of the drill. As shown in Figure 11, tool life was measured in terms of the number of holes drilled and tapped under constant machining conditions and almost the same levels of hardness in the B319.2 and 396 alloys for the M12, M13, M14, M1, M2, and M7 alloys studied. It should be noted that the symbol (+) used in Figure 11 indicates that the tool remained unbroken after the listed number of holes had been drilled and tapped, and could thus have been used for further drilling.

In the drilling tests, the B319.2-M12 alloy containing 0.15%Sn yielded the longest drill life, i.e., twice that of the B319.2-M13 alloy containing 0.5%Bi, and one-and-a-half times that of the B319.2-M14 alloy containing 0.15%Sn + 0.5%Bi. Thus, the addition of 0.15%Sn to the B319.2 alloy has a positive effect on tool life for both the carbide drill and the HSS tap. This extended tool life may be attributed to the size, shape, and distribution of Sn-containing particles. The Sn particles were smaller and more densely distributed in comparison with the Bi particles in the B319.2-M13 alloy. According to the Mg–Sn binary phase diagram, Mg forms the intermetallic compound Mg_2_Sn containing 29 wt% Sn with a melting point at 770.5 °C. Microstructure analysis also showed that Sn does not seem to form the higher-melting intermetallic with Mg in the alloy. The formation of a high-melting compound is undesired from a machining point of view.

In contrast, the presence of 0.5%Bi in the B319.2 and 396 alloys causes a deterioration of tool life (cf., 1101 and 637 holes with 2100 and 2160 holes drilled in the B319.2-M12 alloy containing 0.15%Sn and 396-M1 alloy, respectively). This noticeable reduction in tool life may be explained by the fact that Bi counteracts the modification effect of Sr due to Bi–Mg–Sr interactions that reduce the amount of free Sr available for Si modification; these interactions are expected to take place in the molten state, or during solidification, prior to the precipitation of the eutectic Si. As a result, the Si appears in a coarse, acicular form instead of being fibrous, as shown clearly in Figure 6 and by the eutectic Si particle characteristics in Table 6. If the eutectic silicon structure is coarse, tool life suffers and results in an increase in the rate of tool wear.

In tapping tests, it was found that the B319.2-M12 alloy containing 0.15%Sn showed a higher number of holes tapped (1549 holes), followed by B319.2-M13 containing 0.5%Bi (1500 holes tapped) and by B319.2-M14 containing 0.15%Sn + 0.5%Bi (1235 holes tapped). Thus, the B319.2 alloy containing a combined addition of Sn and Bi shows the lowest number of holes tapped; such an effect may be explained by the fact that Bi has a poor affinity for Sn, and alloys having these two components may not always form the desired low-melting compounds.

### 3.5. Built-Up Edge (BUE) Evolution and Tool Wear Characteristics

As a result of high temperatures, small particles of metal tend to adhere to the edge of the cutting tool, thereby leading to build-up. The presence of the built-up edge (BUE) is capable of inducing a number of effects, including an alteration in tool geometry, thereby reducing the area for the contact of the chip with the rake face. Consequently, the shear plane angle is increased, while the cutting and feed forces are reduced. The fragments of BUE, which are constantly breaking away, leave a very rough surface finish, so that high cutting speeds must be applied if surface quality is important. In practice, the size and shape of the BUE vary greatly with the workpiece material and the prevailing cutting conditions. At higher rates of metal removal, i.e., at a higher speed or feed rate, a BUE will no longer be observed, since the transition from built-up edge to flow zone is strongly influenced by both speed and feed; also, it occurs in the range of cutting conditions commonly encountered in industrial machining operations. The flow zone, described as a thermoplastic shear band, is usually more strongly bonded to the tool than the built-up edge.

**Figure 12:**
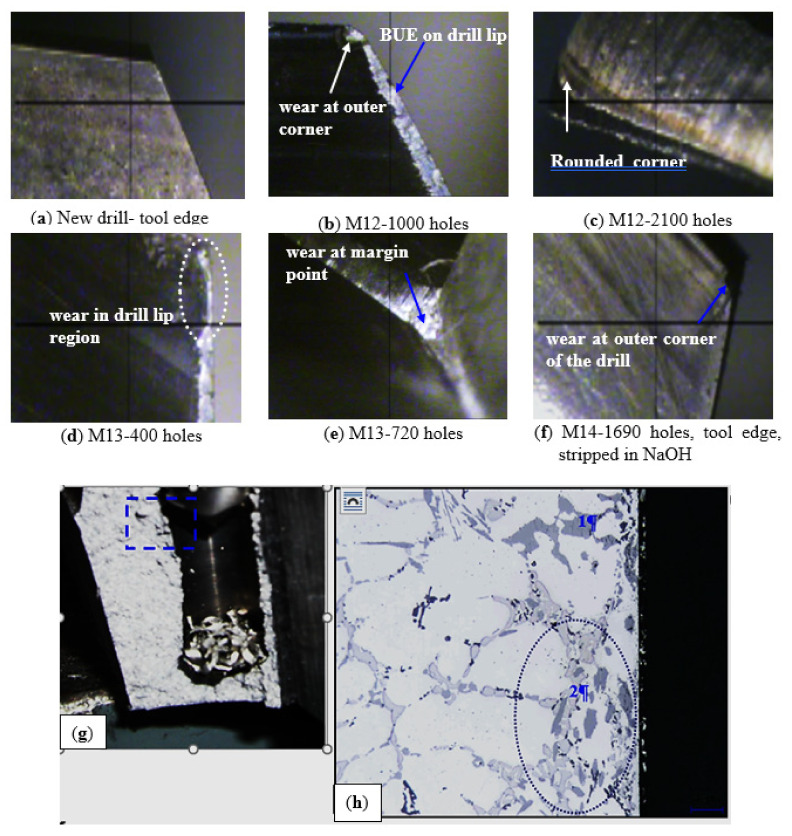
(**a**–**f**) Optical macrographs showing heat build-up and wear on the cutting drill lip and margin point after drilling B319.2 (M12, M13, M14) alloys for different numbers of holes, (**g**) cross-section of a drilled hole in a 396 alloy sample illustrating the area investigated; and (**h**) high-magnification micrograph of (**g**) the drill breakage portion, showing the presence of large Si particles, marked 1, and coarse undissolved Cu-phases, marked 2, in this area.

The tendency of each alloy variation to stick and build up on the cutting edge of the tool was evaluated by high-magnification measurements of the top-view projected area of the actual build-up on the drill. The built-up edge (BUE) width of the tool was also measured after every one hundred drilled holes using a toolmaker’s microscope (TM-505 type) at a magnification of 50×. Figure 12 shows optical images of build-up and wear on the cutting drill lip and margin point after drilling different numbers of holes in the B319.2 alloys.

The build-up measurements reveal that there is little change in the width of the BUE for different numbers of holes except for the alloy containing 0.5%Bi, which showed a slightly lower width of the built-up edge (0.166 mm) compared with those containing 0.15%Sn (0.184 mm) and 0.15%Sn + 0.5%Bi (0.170 mm). Table 8 presents examples of the BUE values for 396 alloys, showing the effect of Fe intermetallics. These results show satisfactory agreement with Jorstad [24], who reported that neither the Sn, nor the Pb, nor the 1%Zn contributed any significant improvement in built-up height during the machining of 380 alloys.

Drill wear has a strong effect on hole quality and dimensional accuracy that can reach a threshold level, thereby causing catastrophic failure of the drill. Excess tool wear is associated with high cutting forces and can damage the workpiece, the fixture, and the machine tool. Basically, there are two main regions for tool wear in a cutting tool, i.e., flank wear on the tool flank face and crater wear on the tool rake face. In this study, however, corner wear instead of flank wear or crater wear was used to predict the tool wear in drilling operations; this is not only because corner wear on the drill is easy to measure but also because drill life is characterized strongly by corner wear on the drill. Figure 12a–c show the state of drill wear at various points in the life of the tool, including: (a) prior to drilling; (b) after the middle holes were drilled; and (c) after the final holes were drilled. It will be seen from these figures that the maximum wear takes place at the outer corner edge, while the minimum wear occurs at or near the point of the tip because the maximum rotational force and the maximum drill-to-workpiece contact occur further away from this point. The outer corner thus tends to become abraded more rapidly, whereas at the point of the tip, little rotational force is experienced and this force is more like “pushing” into the workpiece rather than cutting into it. Figure 12d,e show the effects of bismuth oxide ((BiMg)O) [25,26] on the tool edge, while Figure 12f shows the condition of a failed drill after stripping the adherent workpiece material in a solution of sodium hydroxide (NaOH). As may be seen, wear is visible on the outer cutting corner of the drill. Analysis of the worn drills showed that there is little correlation between the amount of wear and the point of failure, which appears to be governed by the degree of chip/flute clogging.

Figure 12g displays the structure of a 396 base alloy sample (containing 1.2%Cu and 0.25%Mg in addition), corresponding to the portion in which the drill was broken during machining. Figure 12h shows the microstructure of the rectangular area in (g) at high magnification. It will be observed that the segregation of the hard second-phase constituents, which include Al_2_Cu, Al_5_Si_6_Cu_2_Mg_8_, and coarse Si particles as seen in the figure, is detrimental to tool life; thus, if their presence is necessary, it is best that they should be as fine and as dispersed as possible. With this limiting condition in mind, the dominant variables influencing tool life and tool wear in Al–Si alloys are: the morphology of eutectic silicon, the inhomogeneities of the alloy structure, and an interrupted regime of cutting resulting from the coarse undissolved particles.

### 3.6. Chip Characterization

**Figure 13:**
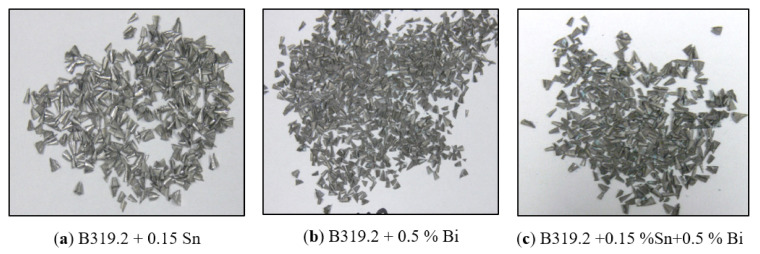
Optical macrographs (**a**–**c**) showing different types of chip obtained for B319.2-M12, B319.2-M13, and B319.2-M14 alloys after drilling the specified number of holes.

The main problems in the drilling of Al cast alloys are considered to be the adhesion, or welding, of the chip on the drill as well as chip/flute clogging. All of these problems are significantly linked to the process of chip formation and chip removal within the flute. The importance of machinability in free-cutting alloys with its concomitant complexity of properties may be defined through a number of decisive criteria involving the size and shape of the chips. Figure 13a–c show the effect of Sn and/or Bi on typical chip formations produced during the drilling of Sr-modified, grain-refined, heat-treated B319.2 alloys. It was found that the fan shape was by far the predominant form, and that it is considered to be the ideal chip for a greater number of drilling applications.

## 4. Conclusions

The following conclusions may be formulated from experiments conducted to study the influence of Sn and Bi additions on the machinability characteristics of Sr-modified, grain-refined, and heat-treated 396 and B319.2 alloys.

1. The formation of the α-Fe phase in the M1 alloy has a beneficial effect on tool life, in that the 396 alloy produces the highest number of holes drilled compared with sludge- or β-Fe containing alloys.
2. The Bi-containing alloys have a detrimental influence on drill life, although they are observed to exhibit lower drilling forces compared with the 396 base alloys.
3. The significant reduction in drill life may be explained by the fact that the presence of 0.5%Bi leads to noticeable coarsening of the Si particles. Moreover, the effectiveness of adding the low-melting-point Bi for the purpose of enhancing machinability is reduced by the loss of Bi in the formation of the high-melting-point Bi_2_Mg_3_ phase.
4. The addition of 0.15%Sn to the 396 alloy has a beneficial effect on the tool life of both carbide drills and HSS taps. Such an effect may be ascribed to the precipitation of β-Sn particles having a low melting point.
5. In drilling tests, the combined addition of 0.15%Sn and 0.5%Bi to the B319.2 alloy improved the alloy’s machinability by lowering the drilling force and moment compared with the B319.2 containing 0.15%Sn alone.
6. The addition of 0.15%Sn to the B319.2 alloy has a beneficial effect on tool life for both carbide drills and HSS taps. During the drilling test, the B319.2-M12 alloy containing 0.15%Sn yielded the longest tool life, namely twice that of the B319.2-M13 alloy containing 0.5%Bi and one-and-a-half times that of the B319.2-M14 alloy containing 0.15%Sn + 0.5%Bi.
7. In tapping tests, however, it was found that the B319.2 containing 0.15%Sn showed the highest number of holes tapped.
8. The presence of 0.5%Bi in the B319.2 alloy causes a certain amount of deterioration in drill life (cf., 1101 holes with 2100 holes drilled in the B319.2-M12 alloy containing 0.15%Sn). This significant reduction in drill life may be explained by the fact that Bi is precipitated in the form of bismuth oxides [(BiMg)O] due to its high affinity for oxygen, especially at high temperatures such as 750 °C.
9. A study of build-up in both 396 and B319.2 alloys coupled with an examination of optical images revealed that there is little change in the width of the BUE for different numbers of holes except in the case of the alloy containing 0.5%Bi, which shows a slightly lower width of the built-up edge than was observed in the alloys under investigation.
10. An examination of worn drills shows that the maximum wear takes place at the outer corner edge, while the minimum wear occurs at or near the point of the tip. It was also found that there is little correlation between the amount of wear and drill failure. This type of failure appears to be governed by the extent of the chip/flute clogging effect.

## Figures and Tables

**Table 1:** Chemical composition of the 396 and B319.2 base alloys.

Alloy	Element (wt%)								
	Si	Cu	Mg	Fe	Mn	Sr	Ti	Al	Mn/Fe
396	10.89	2.24	0.309	0.464	0.492	0.014	0.057	bal.	1.06
B319.2	7.16	3.32	0.29	0.29	0.27	0.019	0.27	bal.	0.95

**Table 2:** Chemical composition of the alloys studied: (Average of three spectrometric analyses).

Alloy Code	Element (wt%)												
	Si	Fe	Cu	Mn	Mg	Zn	Sn	Ti	Bi	Sr	Al	Mn/Fe	S.F
(**a**) B319.2													
M11	7.76	0.30	3.39	0.29	0.37	0.14	-	0.15	0.00	0.018	87.2	0.96	0.9
M12	7.76	0.30	3.39	0.29	0.37	0.14	0.16	0.15	0.00	0.018	87.2	0.96	0.9
M13	7.45	0.29	3.34	0.28	0.38	0.13	0.03	0.15	0.52	0.011	87.3	0.97	0.87
M14	7.33	0.29	3.29	0.28	0.37	0.13	0.15	0.15	0.56	0.019	87.3	0.98	0.86
(**b**) 396													
M1	11.38	0.46	2.22	0.54	0.33	0.0	0.00	0.16	0.00	0.018	84.8	1.17	1.56
M2	11.32	0.46	2.26	0.52	0.34	0.0	0.18	0.17	0.00	0.023	84.6	1.13	1.51
M7	10.97	0.46	2.28	0.59	0.38	0.0	0.00	0.16	0.50	0.019	84.5	1.28	1.64

**Table 3:** Results of hardness measurements for machinability alloys.

Alloy Code	Alloy	HBN (Heat-Treated)
M12	B319.2 + 0.15%Sn	108.08 ± 3.99
M13	B319.2 + 0.5%Bi	111.8 ± 5.52
M14	B319.2 + 0.15%Sn + 0.5%Bi	108.55 ± 3.68
M1	396 + 200 ppm Sr + 0.15%Ti	107.96 ± 3.56
M2	396 + 0.15%Sn	106 ± 3.19
M7	396 + 0.5%Bi	116.96 ± 3.76

**Table 4:** Optimum drilling and tapping conditions [14].

Parameters	Drilling	Tapping
Speed	234.5 m/min or 11,000 rpm	9.57 m/min or 400 rpm
Diameter	Carbide “G” drills 6.5 mm	TiN-coated HSS cutting taps, 5/16-18 UNC-2B
Depth of cut	Depth ≤ 4.5 × D (28.38 mm)	Depth ≤ 3 × D (20.76 mm)
Feed Rate	44 IPM (0.1016 mm/rev)	22.22 IPM (1.41224 mm/rev)
Coolant	Synthetic metalworking fluid concentrate CIMTECH^®^ 310 (5% cutting fluid + 95% liquid)	

**Table 5:** Chemical composition of the observed Mg_2_Sn phase.

Phase	Element	wt%	at%	Calculated Formula	Shape Characteristics	Suggested Formula
Mg–Sn	Mg	30.05	68.01	Mg_2.1_Sn	Tiny particles	Mg_2_Sn
	Sn	68.64	31.81		grey	
	Total	98.69	99.82			

**Table 6:** Silicon particle characteristics for the alloys studied.

Alloy Code	Particle Area (µm^2^)		Particle Length (µm)		Roundness Ratio (%)		Aspect Ratio		Density (Particles/mm^2^)
	Av *	SD **	Av	SD	Av	SD	Av	SD	
(**a**) B319.2 (Al-7%Si)									
M12	7.44	10.6	4.33	3.73	60	18.9	2.07	1.05	12,000
M13	45	70.3	14.3	13.9	31	19.6	3.49	2.43	2000
M14	37.6	59.1	13	12.3	33.7	20.9	3.51	2.48	3100
(**b**) 396 (Al-11%Si)									
M1	4.30	6.51	2.96	2.49	70	17.3	1.77	0.784	28,000
M2	4.72	6.10	3	2.22	65	16.2	1.69	0.681	23,000
M7	48.8	76.8	15.3	13.7	31	19.2	3.54	2.31	2500

* Average value, ** Standard deviation.

**Table 7:** Summary of mechanical properties for the alloys studied.

Alloy Code	BHN (MPa)	YS (MPa)	UTS (MPa)	El (%)
(**a**) 396 alloy (Al-10.8%Si)				
M1	108 ± 3.56	358.10 ± 1.55	394.04 ± 6.27	0.98 ± 0.12
M2	106 ± 3.19	351.65 ± 2.57	390.54 ± 5.73	1.02 ± 0.15
M7	115 ± 3.76	321.39 ± 3.34	360.61 ± 11	0.65 ± 0.14
(**b**) B319.2 (Al-7%Si)				
M12	109 ± 3.68	289.00 ± 2.7	290.99 ± 11	0.626 ± 0.06

**Table 8:** Effects of Fe intermetallics on the width of the built-up edge (BUE) during the drilling process.

Alloy	BUE Width (mm) after Drilling the Specified Numbers of Holes							
No. of Holes Drilled →	180	360	540	720	900	1080	1620	Average
M1 (containing *α*-Fe phase)	0.163	0.162	0.160	0.169	0.179	0.179	0.169	0.168
M3 (containing sludge phase)	0.160	0.158	0.155	0.159	0.150	-	-	0.156
M4 (containing *β*-Fe and *α*-Fe phases)	0.177	0.166	0.170	0.175	0174	-	0.173	0.173

## Data Availability

Data available upon request.
